# *In-vitro *activity of polymyxin B in combination with imipenem, rifampicin and azithromycin versus multidrug resistant strains of *Acinetobacter baumannii *producing OXA-23 carbapenemases

**DOI:** 10.1186/1476-0711-5-10

**Published:** 2006-04-21

**Authors:** David W Wareham, David C Bean

**Affiliations:** 1 Centre for Infectious Disease, Institute of Cell and Molecular Science, Barts and The London, Queen Mary's School of Medicine and Dentistry, London, UK; 2 Department of Medical Microbiology, Barts and The London NHS Trust, London, UK

## Abstract

**Background:**

*Acinetobacter baumannii *has emerged as a major nosocomial pathogen worldwide. Many of the circulating strains exhibit multi-drug resistance remaining consistently susceptible only to polymyxins. *In-vitro *studies have reported that polymyxins combined with carbapenems, rifampicin or azithromycin are synergistic against these strains despite *in-vitro *resistance to these agents alone. The use of antimicrobial combinations have therefore been advocated for the treatment of severe *A. baumannii *infection in man. In order to determine whether such combinations are synergistic against the prevalent clones of multi-drug resistant *A. baumannii *causing infection in the UK, we performed synergy testing against representative isolates using two rapid Etest methods.

**Methods:**

The activity of polymyxin in combination with imipenem, azithromycin or rifampicin was assessed against five strains of multi-drug resistant *A. baumannii *encoding OXA-23 carbapenemases. Synergy studies were performed by Etest-agar dilution and a combined Etest strip method. Synergy was defined as a FICI of ≤ 0.5.

**Results:**

All strains were resistant to β-lactams, carbapenems, quinolones and aminoglycosides but susceptible to polymyxins. Marked synergy was not seen with polymyxin in combination with imipenem, rifampicin or azithromycin against any of the strains. Borderline synergy (FICI = 0.5) was seen against one strain belonging to OXA-23 clonal group 2, using the Etest-agar dilution method only.

**Conclusion:**

*In-vitro *synergy with polymxyin in combination with imipenem, rifampicin or azithromycin is highly strain and method dependent. As reliable synergy could not be demonstrated against the prevalent UK multi-drug resistant strains, use of such combinations should not be used for empirical treatment of these infections in the UK. The optimal treatment for serious multi-drug *A. baumannii *infection and the role of combination therapy should be addressed in a prospective clinical trial.

## Background

*Acinetobacter baumannii *has emerged as an important nosocomial pathogen causing ventilator associated pneumonia, bacteraemia and sepsis in the critically ill. Several strains exhibiting multi-drug resistance have been associated with ongoing outbreaks of infection in intensive care units in London and the South East of England which have proved extremely difficult to treat and control. These strains, designated OXA-23 clone 1 and OXA-23 clone 2 are resistant to all β-lactams (including carbapenems), fluoroquinolones and most aminoglycosides, remaining consistently susceptible only to polymyxin [1]. Many patients have therefore undergone treatment with the polymyxin E formulation, colistin although there is little data on its efficacy in this setting. Colistin has limited tissue penetration when administered intravenously and may not achieve adequate concentrations in important foci of *A. baumannii *infection such as the respiratory tract [2]. It is also only weakly bactericidal at low concentrations versus *A. baumannii in-vitro *[3]. The activity of an antimicrobial agent can sometimes be enhanced by the use of another agent with a different mode of action in combination. Recently polymyxin with imipenem and rifampicin and polymyxin with meropenem, rifampicin or azithromycin were reported as combinations with significant *in-vitro *activity against multi-drug resistant strains of *A. baumannii *[4,5]. We set out to confirm this finding by determining the *in-vitro *activity of polymyxin in combination with imipenem, rifampicin or azithromycin versus epidemic clones of *A. baumannii *producing OXA-23 carbapenemases using an E-test method for the detection of synergy.

## Methods

### Strains

Five multidrug resistant strains of *A. baumannii *known to produce OXA-23 carbapenemases were obtained from the Epidemiological Typing Reference Unit at the Health Protection Agency (HPA, Colindale, UK). The strains were confirmed as *A. baumannii *by biochemical profiling using API 20NE strips (Biomerieux, Marcy d'Etoile, France) and confirmed as resistant to ampicillin, cefotaxime, ceftazidime, ciprofloxacin, piperacillin/tazobactam, gentamicin, amikacin and imipenem by the British Society for Antimicrobial Chemotherapy (BSAC) disc diffusion method [6]. As the OXA 23 clones make up approximately 86% of all carbapenem resistant strains of *A. baumannii *refered to the HPA since 2000 [7] the isolates used in this study are representative of the epidemic UK clones.

### Antimicrobial agents

Polymyxin was obtained from Sigma (Poole, Dorset) and Etest strips from AB Biodisk (Solna, Sweden). All testing was carried out in Isosensitest agar (Oxoid, Basingstoke, UK). The MICs to polymyxin were determined by Etest and agar dilution and the MICs to imipenem, rifampicin and azithromycin by Etest.

### Synergy assays

For synergy screening by Etest – agar dilution, polymyxin was incorporated into Isosensitest agar at 0.25 X the agar MIC for each strain tested. Plates were inoculated with 0.5 McFarland suspensions of each isolate and imipenem, rifampicin or azithromycin Etest strips applied. Following incubation for 24 hrs at 37°C the strips were read and the Etest MICs compared to a series performed in the absence of polymyxin. Synergy screening was also performed using a method employing two Etests applied at right angles to each other, as described by Bonapace *et al*. [8]. Plates were set up with the Etests intersecting at the MIC of each agent tested, inoculated and incubated as above. Fractional inhibitory concentration indices (FICIs) were calculated as MIC of drug X (in combination) / MIC of drug X alone + MIC of drugY (in combination) / MIC of drugY alone. Synergy between agents was defined as a FICI ≤ 0.5.

## Results

**Figure 1:**
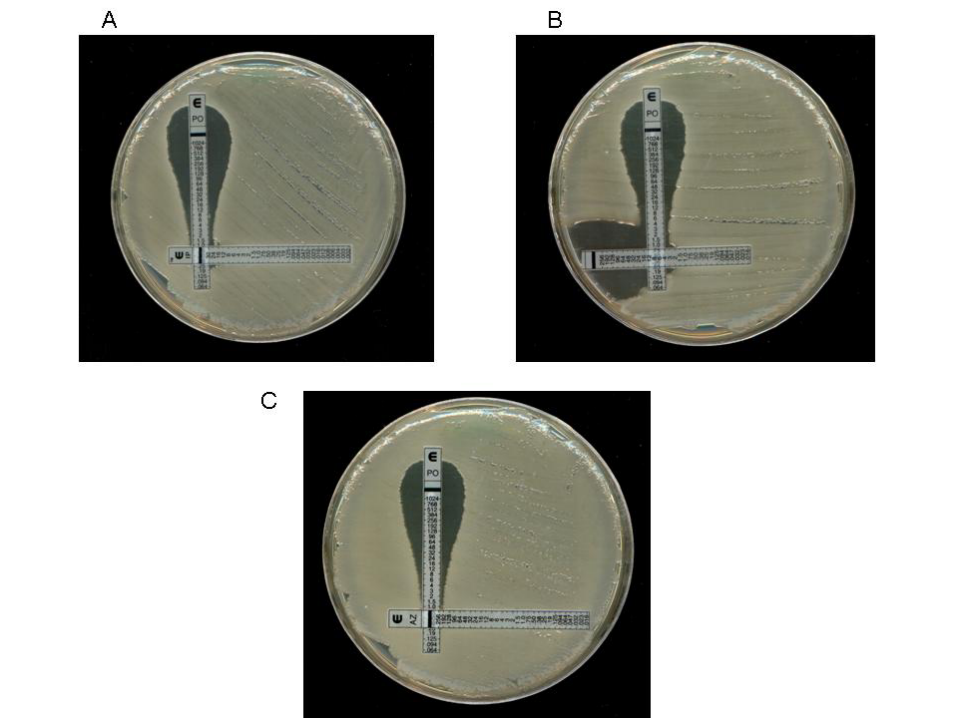
Activity of polymyxin B in combination with imipenem, rifampicin and azithromycin versus a multidrug resistant A. *baumannii *OXA-23 clones using 2 Etest method. A) Polymyxin and imipenem versus OXA-23 clone 2, B) Polymyxin and rifampicin versus OXA-23 clone 2, C) Polymyxin and azithromycin versus OXA-23 clone 1

Four of the isolates were members of OXA-23 clone 1 and the other was a member of OXA-23 clone 2. The MIC's of all five isolates to imipenem, rifampicin, polymyxin and azithromycin are shown in Table 1. Using the Etest-agar dilution method imipenem in combination with polymyxin resulted in a reduction in the imipenem MIC for two out of five strains with synergy (FICI = 0.5) observed for the OXA-23 clone 2 strain. Rifampicin in combination with polymyxin led to reduced MICs with all strains by Etest-agar dilution but this was not synergistic for OXA-23 clone 1 isolates (FICI = 0.625 - 0.75) and only borderline for the OXA-23 clone 2 strain (FICI = 0.5). Synergy was not observed using polymyxin in combination with azithromycin. Using two Etests to screen for synergy, no reduction in imipenem, rifampicin or azithromycin MIC was observed for any of the OXA-23 clone 1 isolates. A reduction in the rifampicin MIC to 2 mg/L and the polymyxin MIC to 0.125 mg/L was seen for the OXA-23 clone 2 isolate when these agents were used in combination (Figure 1). Likewise the azithromycin MIC fell to 12 mg/L and the imipenem MIC to 24 mg/L when used in combination with polymyxin. None of these combinations however resulted in synergy versus OXA-23 clone 2 (FICI ≥ 1).

**Table 1:** Characteristics of A. *baumannii *isolates

Strain	Clonal Group	Polymyxin MIC (mg/L)	Imipenem MIC (mg/L)	Rifampicin MIC (mg/L)	Azithromycin MIC (mg/L)
1	OXA-23 clone 1	0.25	12	4	32
2	OXA-23 clone 1	0.25	>32	4	>256
3	OXA-23 clone 1	0.25	>32	4	>256
4	OXA-23 clone 1	0.25	>32	4	>256
5	OXA-23 clone 2	0.25	>32	4	24

## Discussion

The problem of multi-drug resistance in clinical isolates of *A. baumannii *has led to widespread use of polymyxins for the treatment of severe infections with this organism. Several studies have evaluated the ability of other agents to produce synergy when combined with polymyxin *in-vitro *[4,5]. The aims of the present study were therefore two-fold; firstly to determine the *in-vitro *efficacy of polymyxin in combination with other agents against epidemic UK *A. baumannii *clones, and secondly, to evaluate the use of rapid methods of testing antibiotic synergy which could be employed in routine clinical practice.

We were unable to show synergy with either imipenem, rifampicin or azithromycin in combination with polymyxin for any of the OXA-23 clone 1 isolates. For the OXA-23 clone 2 isolate synergy was observed (FICI = 0.5) with polymyxin in combination with rifampicin or imipenem The clinical relevance of this synergy can be debated as the MIC to imipenem in combination with polymyxin was still 8 mg/L which is still above the imipenem break point of 4 mg/L. As the isolates used in this study are the prevalent multi-drug resistant strains in the United Kingdom this has implications for the use and selection of combination antimicrobial regimens for empirical treatment of these infections. In the study by Timurkaynak *et al *testing for synergy between colistin, rifampicin, meropenem and azithromycin [5], synergy with colistin and meropenem was seen versus two isolates. The five strains used in this study however were not uniformly resistant to carbapenems as those seen in the UK (meropenem MIC 1 – 64 mg/L). Amongst the isolates where synergy was demonstrated with meropenem the isolates had meropenem MICs of 2 and 4 mg/L. These would be considered sensitive using BSAC (sensitive ≤ 4) [6] or intermediate (resistant =16) by the US CLSI [9] recommendations on meropenem breakpoints, suggesting that synergy can be obtained with carbapenems in combination with colistin if resistance to carbapenems is only at a low level. Hence in our study using isolates with high level carbapenem resistance (MIC > 32 mg/L) synergy was not observed using imipenem in combination with polymyxin.

In contrast to our findings, a study comparing checkerboard FICI determination with time kill assays on eight *A. baumannii *isolates with high level imipenem resistance (MIC = 32 mg/L) did demonstrate synergy between polymyxin B in double and triple combinations with imipenem and rifampicin [4]. However, in this instance an incorrect interpretation of ∑FICs lead to erroneous conclusions being drawn. Although synergy versus all of the isolates with the triple combination and against seven with double combinations was reported, a FICI of ≤ 1 was used as indicative of synergy.

This definition is generally considered too relaxed [10], and if the more stringent definition (FICI ≤ 0.5) is applied only three of the double and two of the triple combinations would be considered synergistic, although, interestingly one of these is between polymyxin B and imipenem.

Whilst most synergy studies have been performed using checkerboard or time kill assays these methods are too time consuming and technically challenging for routine clinical testing. Our decision to use Etest was therefore based on its ease of use and availability to routine clinical laboratories. As Etests deliver a controlled concentration gradient of antibiotic into the agar, errors in the preparation of the media and antibiotic dilutions required for synergy testing using checkerboard and time kill methodology are avoided. In a comparison of Etest, time kill and checkerboard assays using ten strains of *Acinetobacter baumannii *and the antibiotics piperacillin, trovofloxacin, cefipime and tobramycin, Etest was noted to be more conservative in detecting synergy than either of the other two methods [8]. Although this suggests that Etest may not be effective in detecting weak synergy, it is debatable what, if any, clinical significance detection of such activity may have. The ability of Etest to rapidly detect only marked synergy is likely to be far more useful in aiding the selection of antimicrobials combinations for the treatment of multidrug resistant infections. A novel combination gradient test, Xact™ has recently been developed by AB Biodisk. This uses perpendicularly aligned concentration gradients of antibiotics embedded in a plastic carrier, for direct application to an agar surface. A colistin – rifampicin Xact™ test was used to quantify synergy between colistin and rifampicin against six strains of carbapenem resistant *A. baumannii *[11]. Synergy was reported against these strains and there was good concordance between checkerboard and the Etest-agar dilution method. The Xact™ test may therefore be a useful method for screening for strain dependent synergy of agents in combination against clinical isolates of *A. baumannii*.

There are very few studies of the efficacy of combination regimens identified by *in-vitro *synergy screening in the treatment of multi-drug resistant *Acinetobacter *infections. A favourable outcome based on eradication of *A. baumannii *from clinical samples, was reported for 26 patients with *A. baumannii *ventilator associated pneumonia, bacteraemia and meningitis when colistin was used in combination with rifampicin [12]. This study did not however assess patient outcome or compare the efficacy of colistin alone. No data was reported on the *in-vitro *activity of the agents in combination and whether there was *in-vitro *synergy against the infecting strains by any method. It is therefore difficult to determine whether the reported efficacy should be ascribed to colistin alone, true *in-vivo *synergy between colistin and rifampicin or some unrelated effect of rifampicin such as immunomodulation. As we have not been able to demonstrate consistent synergy between polymyxin and rifampicin against isolates in the UK exhibiting high level carbapenem resistance we would not advocate empirical use of such a combination unless this can be shown on a strain by strain basis. The optimal treatment of multi-drug resistant *Acinetobacter *infections should now be addressed via a well designed randomised controlled clinical trial.

## Conclusion

The optimal treatment of multi-drug resistant *Acinetobacter *infections remains controversial. Despite reports of enhancedactivity of polymyxin combined with imipenem, rifampicin or azithromycin we were unable to detect reliable *in-vitro *synergy between polymyxin B and any of these agents versus epidemic strains of *A. baumannii *with high level carbapenem resistance due to OXA-23 carbapenemases. At present we do not advocate empirical use of these combination regimens in the UK until more clinical data on their efficacy is available.

## Competing interests

The author(s) declare that they have no competing interests.

## Authors' contributions

Both authors contributed equally to the laboratory studies, drafting and editing of the manuscript.

**Table 2:** Polymyxin synergy with imipenem, rifampicin and azithromycin by Etest – agar dilution

Strain	Imipenem MIC with polymyxin 0.25 × MIC (mg/L)	FICI	Rifampicin MIC with polymyxin 0.25 × MIC (mg/L)	FICI	Azithromycin MIC with polymyxin 0.25 x MIC (mg/L)	FICI
1	6	0.75	1.5	0.625	24	1
2	>32	1.00	1.5	0.625	>256	1
3	>32	1.00	2	0.75	>256	1
4	>32	1.00	1.5	0.625	>256	1
5	8	0.5	1	0.5	24	1
